# Medical Jurisprudence, Insanity and Toxicology

**Published:** 1903-10

**Authors:** 


MEDICAL JURISPRUDENCE, INSANITY AND TOXICOLOGY.—By Henry C. Chapman, M. D., Professor of Institutes of Medicine and Medical Jurisprudence in the Jefferson Medical College, Philadelphia. Third edition, thoroughly revised, greatly enlarged, and entirely reset. Handsome 12mo volume of 329 pages, fully illustrated, including four colored plates. Philadelphia, New York, London. W. B. Saunders & Co. 1903. Cloth, $1.75 net.

This manual contains the entire course of lectures delivered annually by Dr. Chapman to the students of Jefferson Medical College, which are based on the author's six years experience as Coroner's Physician of the city of Philadalphia. It should serve to stimulate a greater interest in the study of this important branch of medicine. In this, the third edition, revised and enlarged, nothing of importance to either the physician or lawyer has been left out.

